# Chemoselective Polymerization Control: From Mixed-Monomer Feedstock to Copolymers**

**DOI:** 10.1002/anie.201309575

**Published:** 2014-01-22

**Authors:** Charles Romain, Charlotte K Williams

**Affiliations:** Department of ChemistryImperial College London, London SW7 2AZ (UK)

**Keywords:** homogeneous catalysis, chemoselectivity, polycarbonates, polyesters, ring-opening polymerization

## Abstract

A novel chemoselective polymerization control yields predictable (co)polymer compositions from a mixture of monomers. Using a dizinc catalyst and a mixture of caprolactone, cyclohexene oxide, and carbon dioxide enables the selective preparation of either polyesters or polycarbonates or copoly(ester-carbonates). The selectivity depends on the nature of the zinc–oxygen functionality at the growing polymer chain end, and can be controlled by the addition of exogeneous switch reagents.

A key challenge for polymer science is to control the polymer sequence from mixtures of monomers. Sequence-controlled multiblock copolymers are of high interest, owing to their enhanced properties and their potential for designed functionality. Whereas Nature exerts sophisticated sequence control, for example, in peptide synthesis, synthetic methods are far less advanced. Generally, when using mixed-monomer feedstocks, the relative reactivities of the monomers, determined from homopolymerization experiments, are used to predict the sequence of the copolymer, that is, kinetic control.^1^ Recently, this has been elegantly exploited using donor–acceptor monomers, to prepare sequence-controlled block copolymers.^2^ Herein, we present a novel chemoselective control mechanism that is applicable to both the ring-opening polymerization (ROP) of lactones and the ring-opening copolymerization (ROCOP) of epoxides and carbon dioxide to yield copoly(ester-carbonates) (Figure 1).

**Figure 1:**
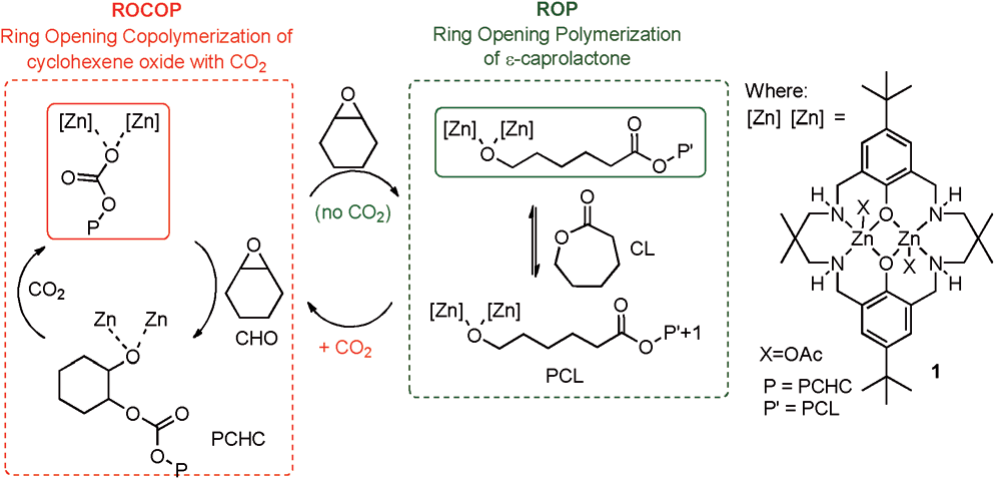
ROP and ROCOP, the switch mechanisms, and the structure of catalyst **1**.

Polyesters and polycarbonates are currently produced on a large scale (>5 Mt/annum), mostly by condensation polymerization and using petrochemicals.^3^ Such syntheses are hampered by precise monomer stoichiometry requirements, thermodynamic constraints, slow rates, and a total lack of polymerization control. These factors complicate, and even prevent, the preparation of copolymers.^3^ In contrast, the ring-opening polymerization (ROP) of lactones^4^/cyclic carbonates^5^ and the ring-opening copolymerization (ROCOP) of epoxides with CO_2_^6^/anhydrides^7^, ^8^ (Figure 1) are much more desirable syntheses. They are much faster and thermodynamically more favorable than condensation routes. Furthermore, they are highly controlled, yielding polymers of predictable molecular weights with narrow polydispersity indices. As such, they are the syntheses of choice for copolymers. Furthermore, several lactones, epoxides, and carbon dioxide can be sourced from renewable resources. Thus, the development of ROP and ROCOP is of high importance for improved, versatile, and sustainable routes to these commodity polymers.^4^–^7^, ^8^

Both the ROP of cyclic lactones and the ROCOP of epoxides/CO_2_ require catalysts; the most successful of these are homogeneous metal complexes.^4^, ^6^ Curiously, there is very little overlap between the catalysts applied for these two polymerizations: only a few homogeneous complexes can catalyze both processes, almost always independently.^9^ There are two intriguing hints in the literature that combined processes may be feasible, using either heterogeneous zinc glutarate or a β-diketiminate zinc catalyst, but insights into how to combine the reactions are limited.^10^ Indeed, even using multiple catalysts there are only a handful of reports of both ROP and ROCOP.^8^, ^11^ Our group has prepared ABA triblock copolymers by ROCOP, using a dizinc catalyst, followed by intermediate polymer purification and subsequent lactide ROP using an yttrium catalyst.^12^ Darensbourg et al. produced AB and ABA type polymers by tandem catalysis using a cobalt ROCOP catalyst, followed by a DBU ROP catalyst.^8^, ^13^ Although elegant, this method requires careful balancing of the rates of the catalysts, and complexes which will not react with each other.

Here, a straightforward strategy to switch between ROP and ROCOP is presented (Figure 1). The method applies exogeneous switch reagents to direct the polymerization pathway. It enables a single catalyst (**1**) to be used for both ROP and ROCOP, both sequentially and in one pot. It obviates the need for intermediate polymer isolation, complex rate balancing or compatibility testing.

Dizinc complex **1** is an efficient ROCOP catalyst, using cyclohexene oxide (CHO) and CO_2_.^14^ **1** operates under CO_2_ pressures as low as 1 bar and is highly selective, producing poly(cyclohexene carbonate) (PCHC) with a high fidelity of carbonate repeat units (>99 %). Given the strong precedent for zinc catalysts in lactone ROP,4c **1** was tested for the ROP of caprolactone (CL). However, **1** was completely inactive, even under forcing conditions (80 °C, neat CL, with/without alcohol; Table 1; see also Table S1 in the Supporting Information). This appears to be a thermodynamic limitation, as even reaction for extended periods (16 h, >48 half-lives; see below) failed to yield polycaprolactone (PCL). Catalyst deactivation is ruled out, as **1** can be switched on for ROP by addition of 10 mol % (vs. CL) of an epoxide (Table 1, entry 2). Thus, CL ROP using **1** with 10 % added epoxide (CHO) at 80 °C proceeded to complete conversion (>99 %) within 2 h, yielding only PCL (Figure 2, RHS). The polymerization was well controlled; the PCL *M_n_* (21 000 g mol^−1^, PDI: 1.4) was in excellent agreement with that predicted *M*_*n*calc_=22 000 g mol^−1^). The epoxide switch reagent can be added either in low quantities (10 %) or in excess, for example, as the polymerization solvent (Table 1, runs 3–6).Where CHO is the solvent (at >9×conc. of CL), the PCL shows a reduced *M*_n_ owing to chain-transfer reactions with residual water/cyclohexane diol (0.1 mol % vs. CHO); equivalent reactions are common to catalysts in this field.14c, 15

**Figure 2:**
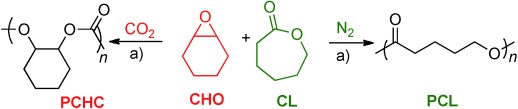
Chemoselective polymerization control. a) **1**/CHO/CL=1:900:100, 80 °C, 16 h. Gases (CO_2_ or N_2_) are added to 1 bar total pressure.

**Table 1:** The formation of polyesters, polycarbonates, and copolymers using 1.

Run	CHO	CL	CO_2_	*t* [h]	% conv. [%][a]	Polymer	*M*_n_ (PDI) [g mol^−1^][b]
1	–	500	–	16	–	–	–
2[c]	20	200	–	2	>99	PCL	11 780 (1.41)
3	900	100	–	2	>99	PCL	6020 (1.24)
4	900	100	1	15	12	PCHC	1040 (1.08)
5[d]	900	100	1	21	>99 (CL) 53 (CHO)	PCL-PCHC	4810 (1.38)
6[e]	450	50	1	6.5	6 (CHO) >99 (CL)	PCHC-PCL	3490 (1.48)
7[e]	450	50	1	4 2	10 (CHO) >99 (CL)	PCHC PCHC-PCL	560 (1.29) 2350 (1.49)

[a] Determined by ^1^H NMR spectroscopy from the normalized integrals for resonances from CL (4.23 ppm), PCL (4.05 ppm), CHO (3.11 ppm), and PCHC (4.73–4.54 ppm).

[b] Determined by SEC in THF, with a correction factor of 0.56 for PCL, as described in Ref. 16.

[c] Polymerization in toluene, [CL]_0_=1 M.

[d] CO_2_ added after complete consumption of CL (1 h), as monitored by ATR-IR spectroscopy.

[e] CO_2_ removed after 3.5 h by application of six vacuum purge/nitrogen flush cycles.

The switch reaction involves attack by a zinc acetate group from **1** on the cyclohexene oxide, forming a zinc alkoxide intermediate. The ATR-IR spectrum of this intermediate (Figure S3) matches theoretical predictions and spectroscopic data.14d The zinc alkoxide intermediate is the only product formed even when using excess epoxide, that is, **1** does not catalyze any homopolymerization of cyclohexene oxide. Together, the data show that **1** (a zinc carboxylate species) cannot initiate CL ROP, whereas the zinc alkoxide intermediate can initiate. Furthermore, the zinc acetate group only undergoes a single ring-opening reaction with CHO. Thus, the cyclohexene oxide switches on the ROP of CL. Efficient metal carboxylate initiators for cyclic ester ROP are scarce.4c For example, [(bdi)Zn(OAc)] (bdi=β-diiminate) shows very low activity for lactide ROP in contrast to [(bdi)Zn(O*i*Pr)], which is highly active.9b In order to establish the generality of the switch, Zn(OAc)_2_ was tested with/without added CHO for CL ROP (Table S1). Without any CHO, there is negligible polymerization, however, adding just CHO (10 mol %), results in complete conversion within 15 h. Furthermore, adding CHO (10 mol %) to CL ROP using [(bdi)Zn(OAc)] yielded PCL in 1 h. The epoxide switch is expected to be general; it could enable ROP using other metal carboxylates. This is desirable, as they are less sensitive than currently applied metal alkoxides/amides.

With an efficient catalyst for either ROCOP or ROP, the development of a one-pot procedure enabling both polymerizations was desirable (Figure 2). The reaction of **1** with all reagents simultaneously yielded only polycarbonate (PCHC), that is, only ROCOP occurred (Figure 2 RHS, Table 1). The ^1^H NMR spectrum of the crude product showed only PCHC formation, with CL remaining unreacted (Figure S4). The polymerization was monitored using in situ ATR-IR spectroscopy (Figure 3). As the reaction progressed, there was a continual increase in PCHC formation (1275 cm^−1^). In contrast, there was no change in either PCL or CL concentrations (1374 and 694 cm^−1^, respectively). In order to rationalize this selectivity, the CL ROP was investigated with CHO (10 mol %), under an atmosphere of CO_2_ (1 bar). The polymerization was inhibited: there was no formation of PCL. This is proposed to be due to rapid insertion of CO_2_ into the zinc alkoxide bond, coupled with the inability of the zinc carbonate to initiate ROP, by analogy to the previous findings using **1** (Figure 4). The rate law for ROCOP using **1**, shows a zeroth order in carbon dioxide (1–40 bar), which is consistent with the rapid insertion of CO_2_ into zinc alkoxide bonds.14c Thus, CO_2_ inserts more rapidly into the zinc alkoxide bond than the rate of CL ROP (Figure 4). At first sight this could be counterintuitive, particularly if only the turn-over frequencies are considered: the TOFs for ROCOP (ca. 7 h^−1^) are significantly lower than those for CL ROP (ca. 100 h^−1^; Table S2).

**Figure 3:**
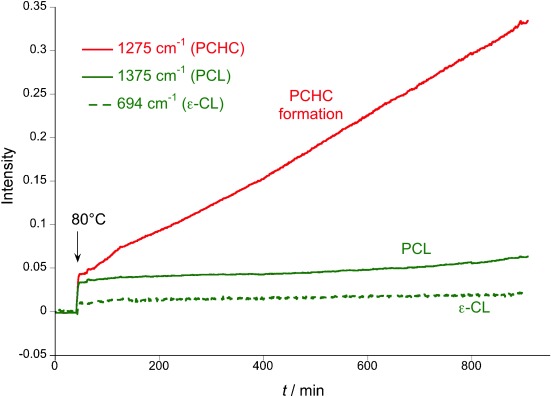
Changes in the intensity of IR resonances (normalized) during the reaction in Figure 2, LHS (Table 1, run 3). The mixture of CL and CHO under CO_2_ (1 atm) shows only the formation of PCHC, with no PCL formation.

**Figure 4:**
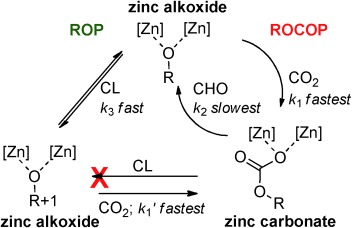
Chemoselective polymerization control, illustrating the importance of the zinc-oxygen chain end groups. The selectivity can be rationalized if the relative rates are: *k*_1_≈*k*_1′_>*k*_3_≫*k*_2_. Where [Zn] [Zn] is defined in Figure 1 and R=growing polymer chain (PCHC/PCL/copolymer).

The explanation lies in the relatively faster rate of the pre-rate-determining step (CO_2_ insertion) which ensures that, from a mixture of monomers, catalyst **1** selects for ROCOP. Another finding is that the nature of the zinc chain end group controls selectivity, even from a mixture of different monomers. The zinc alkoxide chain end group catalyzes either ROCOP or ROP, whereas the zinc carboxylate/carbonate chain end groups only catalyze ROCOP. We have termed this unusual phenomenon: chemoselective polymerization control (an illustration of the key concepts is provided in Figure 4).

A logical next step was to investigate **1** as a catalyst for sequential ROP and ROCOP (Table 1, runs 5–6) by exploiting this chemoselective polymerization control. First, the ROP of CL (100 equiv) was investigated, using **1** dissolved in excess cyclohexene oxide (900 equiv). This resulted in quantitative formation of PCL (*M*_n_=4100 g mol^−1^, PDI=1.4) in less than 1 h. Figure 5 illustrates the in situ ATR-IR data and shows changes to the intensities of the resonances consistent with efficient CL ROP (1750 and 694 cm^−1^) and PCL formation (1420 cm^−1^). Next, carbon dioxide (1 bar total pressure) was added: ROCOP began immediately, leading to PCL-PCHC formation (evidenced by the increase in the intensity of the signal at 1237 cm^−1^). During the next 20 h, the resonances due to PCL and CL (1420 and 694 cm^−1^, respectively) did not change significantly. The intensities of the resonances at 1750 and 1237 cm^−1^ are affected by both CL/PCL and PCHC concentrations (Figure S5), but the dominant influences are apparent. Size-exclusion chromatography (SEC) analysis (53 % CHO conversion, *M*_n_=4800, PDI=1.28) shows only a slight increase in *M*_n_ compared to the PCL formed after 1 h (Figure S6). The *M*_n_ determined by SEC is calibrated against polystyrene standards because the correction factors are unknown for the copolymers: this calibration issue is likely in part responsible for the smaller increase in *M*_n_ than might be expected from the monomer conversions. The ^1^H NMR spectrum (Figure S7) confirms the expected copolymer composition, with relative intensities of carbonate and ester signals in the expected ratios given the monomer conversions. The carbonyl region of the ^13^C{^1^H} NMR spectrum (Figure S8) indicated block copolymer formation, with signals due to PCL (174 ppm) and PCHC (154 ppm) only. There were no intermediate peaks, which is consistent with a lack of copolymer scrambling reactions, for example, transesterification/carbonation.

**Figure 5:**
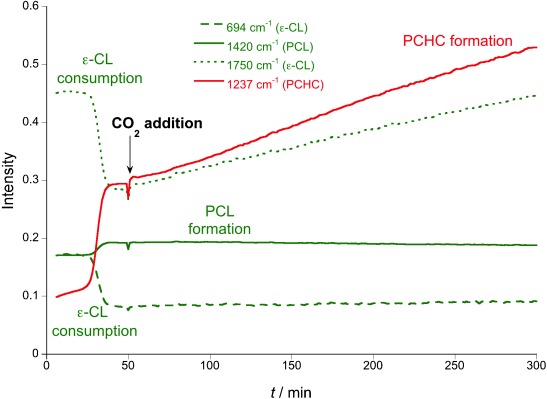
Changes to the intensity of IR resonances during PCL-PCHC formation (Table 1, run 5). The plot shows the ROP of CL, the addition of CO_2_, and the ROCOP of CHO/CO_2_.

The reverse order of monomer addition was also investigated, that is, firstly ROCOP, then ROP. This was achieved by mixing **1** with excess CHO, CL, and CO_2_ (1 bar), so as to initiate ROCOP and produce PCHC. In situ ATR-IR spectroscopy (Figure 6) showed PCHC formation (1280 cm^−1^), with PCL/CL signals remaining constant (1420 and 694 cm^−1^, respectively). After 3.5 h, the CO_2_ was purged from the reaction, by application of six vacuum purge/nitrogen flush cycles over a period of 15 min, after which the intensity of the CO_2_ resonance (2340 cm^−1^) decreased to near zero. The CO_2_ atmosphere was replaced with N_2_, leading to rapid CL ROP. After this time, there was no significant change to the PCHC signal (1280 cm^−1^).

**Figure 6:**
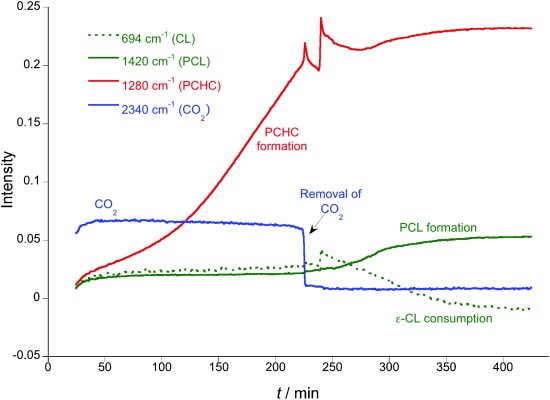
Changes to the intensity of IR resonances for PCHC-PCL formation (Table 1, run 6). The plot shows the ROCOP of CHO/CO_2_, the removal of CO_2_ and the ROP of CL.

The reaction was repeated with aliquot removal for SEC analysis (Table 1, run 7); thus after 4 h there was ca. 10 % PCHC formation with an *M*_n_ of 530 g mol^−1^ (PDI=1.29; Figure S9) and after a further 2 h, there was >99 % conversion into PCL-PCHC and the *M*_n_ had increased to 2350 g mol^−1^ (PDI=1.49; Figure S9). Thus, the reverse monomer addition also enables one-pot sequential ROCOP and ROP, and yields PCHC-PCL (polycarbonate-ester). In this case, it is important to remove the excess CO_2_, so that the zinc carbonate species can react with a further equivalent of CHO to generate the zinc alkoxide species required for CL ROP.

In summary, a means to bridge between two commonly applied polymerization pathways, ring-opening polymerization and ring-opening copolymerization, is described, which enables the selective synthesis of polyesters, carbonates, and copoly(ester-carbonates). The two pathways are bridged by the addition of exogeneous switch reagents, either epoxide or carbon dioxide. The study also reveals that the chemical nature of the zinc-polymer chain end group plays a central role in controlling which monomer(s) are polymerized from a mixture. Such chemoselective polymerization control is unusual, yet the principles uncovered here are expected to apply more generally, including to other epoxides, heterocumulenes, and lactones. Future studies will focus on exploiting it to prepare a range of new polymers/copolymers.
