# Supplementary material for: Chemoselective Polymerization Control: From Mixed-Monomer Feedstock to Copolymers
Source: Angew Chem Int Ed Engl. 2014 Jan 22;53(6):1607–10. doi: 10.1002/anie.201309575 (PMC4232277; doi:10.1002/anie.201309575)
Supplement: Supplementary file 1 [file anie0053-1607-sd1.pdf]

Angewandte Chemie
Eine Zeitschrift der Gesellschaft Deutscher Chemiker

Supporting Information

© Wiley-VCH 2014

69451 Weinheim, Germany

Chemoselective Polymerization Control: From Mixed-Monomer Feedstock to Copolymers**

*Dr Charles Romain and Charlotte K. Williams**

anie_201309575_sm_miscellaneous_information.pdf

Supporting information for

Pages S1-3: Experimental Section

Page S4: Table S1

Page S5: Table S2

Page S6: Fig. S1

Pages S7: Fig. S2, S3

Page S8: Fig. S4, S5

Page S9: Fig. S6, S7

Page S10: Fig. S8

Page S11: Fig. S9

Experimental Section

General procedures

The catalyst $[LZn_2(OAc)_2]$ (**1**) and [(BDI)Zn(OAc)] were synthesized employing the previously published methods.[i] All solvents and reagents were purchased from commercial sources (Aldrich and Merck) and used as received, unless stated otherwise. Toluene was distilled from sodium and stored under nitrogen. ε-caprolactone (ε-CL) was dried over calcium hydride and store under nitrogen. Cyclohexene oxide was fractionally distilled, over calcium hydride, prior to use and stored under nitrogen. Research grade CO_2 for copolymerization reactions was purchased from BOC (Linde Gas). ^1H and ^{13}C NMR spectra were measured in $CDCl_3$ on a Bruker AV-400 spectrometer. *In situ* ATR-FTIR measurements were performed on a Mettler-Toledo ReactIR 4000 spectrometer equipped with a MCT detector and a silver halide DiComp probe. Size exclusion chromatography (SEC) data were collected using a Polymer Laboratories PL GPC-50 instrument (Polymer Laboratories Mixed D columns) with THF as the eluent, at a flow rate of 1 mL min^{-1} at 40°C and narrow M_w polystyrene standards for calibration.

Synthesis of PCL, in the presence of 10 mol. % of CHO (Table 1, Entry 2)

Cyclohexene oxide (25 μL, 0.25 mmol), caprolactone (277 μL, 2.5 mmol), **1** (10 mg, 12.5 μmol) and toluene (2.5 mL) were added to a screw cap vial in the glovebox. The vessel was heated at 353 K, with constant stirring, for 2 h. A sample of the crude product was analyzed by ^1H NMR spectroscopy, to determine the conversion. The volatile components were removed, *in vacuo,* to yield the product as a white powder. M_n = 21,040 g/mol, PDI = 1.41

Synthesis of PCL with CHO as solvent (Table 1, Entry 3)

Cyclohexene oxide (1.1 μL, 0.11 mmol), caprolactone (139 μL, 1.25 mmol), **1** (10 mg, 12.5 μmol) were added to a Schlenk tube. The vessel was heated at 353 K, with constant stirring, for 2 h. A sample of the crude product was analyzed by ^1H NMR spectroscopy to determine the conversion and selectivity. Unreacted reagents (CHO and ε-CL) were subsequently removed, *in vacuo,* to yield the product as a white powder. M_n = 6,020 g/mol, PDI = 1.24

Synthesis of PCHC, in the presence of CL (Table 1, Entry 4):

Cyclohexene oxide (1.1 mL, 10.75 mmol), caprolactone (138.6 μL, 1.2 mmol) and **1** (10 mg, 12.5 μmol) were added to a Schlenk tube. The vessel was de-gassed, then 1 bar of CO_2 was added and the solution allowed to stir for 30 min at 298 K. The vessel was then heated at 353 K, under a constant pressure of CO_2 at 1 bar, for 15 h. The crude reaction mixture was analysed using ^1H NMR spectroscopy to determine the conversion and selectivity. Then, the unreacted monomers were removed *in vacuo* to yield the polymer product. M_n = 1,040 g/mol, PDI = 1.08

Synthesis of poly(caprolactone-co-cyclohexylenecarbonate) (Table 1, Entry 5)

Cyclohexene oxide (2.2 mL, 21.5 mmol), ε-caprolactone (277 μL, 2.5 mmol) and **1** (20 mg, 25.0 μmol) were added to a Schlenk tube. The vessel was heated at 353 K, under N_2, for 1 h, then de-gassed and 1 bar of CO_2 was added. The vessel was

heated for 20 h. A sample of the crude product was analysed by ^{1}H NMR spectroscopy to determine the conversion and selectivity. Any unreacted monomers were removed, *in vacuo,* to yield the product as a white powder. M_n = 4,810, PDI = 1.28.

Synthesis of poly(cyclohexylenecarbonate-co-caprolactone) (Table 1, Entry 6)

Cyclohexene oxide (2.3 mL, 22.5 mmol), ε-caprolactone (277 μL, 2.5 mmol), and **1** (40 mg, 50.0 μmol) were added to a Schlenk tube. The vessel was degassed at 298 K, then CO_2 was added. The vessel was left under a CO_2 atmosphere, at 298 K, for a few minutes and was then heated to 353 K, with continuous reaction stirring, for 3.5 h. Then, the CO_2 was removed from the reaction *via* 6 vacuum-nitrogen cycles, over a period of 15 min. The vessel was maintained at 353 K for 3 h. A sample of the crude product was analysed by ^{1}H NMR spectroscopy to determine the conversion and selectivity. Any unreacted monomers were removed, *in vacuo,* to yield the product as an oily white wax. M_n = 3,490 g/mol, PDI = 1.48.

Synthesis of poly(cyclohexylenecarbonate-co-caprolactone) (Table 1, Entry 7)

Cyclohexene oxide (2.3 mL, 22.5 mmol), ε-caprolactone (277 μL, 2.5 mmol), and **1** (40 mg, 50.0 μmol) were added to a Schlenk tube. The vessel was degassed at 298 K, then CO_2 was added. The vessel was left under a CO_2 atmosphere, at 298 K, for a few minutes and was then heated to 353 K, with continuous reaction stirring, for 4 h. Then, the CO_2 was removed from the reaction *via* 6 vacuum-nitrogen cycles, over a period of 15 min. The vessel was maintained at 353 K for 2 h. A sample of the crude product was analysed by ^{1}H NMR spectroscopy to determine the conversion and selectivity. Any unreacted monomers were removed, *in vacuo,* to yield the product as an oily white wax. M_n = 2,349 g/mol, PDI = 1.49.

Table S1: Different conditions for CL ROP using zinc acetate complexes as catalysts. The catalyst structures are illustrated below for reference.

Entry	Catalyst	CL (eq.)	^{i}PrOH (eq.)	CHO (eq.)	Solvent	T (°C)	t (h)	Conv. (%)	$M_{n\mathrm{exp}}$ [a] (PDI) (g/mol)
1	**1**	500	4	-	Neat	80	3.5	0	-
2	**1**	200	-	20	Toluene[b]	80	2	> 99	11,780 (1.4)
3	$Zn(OAc)_2$	100	-	10	Toluene[c]	100	15	> 99	32,970 (1.82)
4	$Zn(OAc)_2$	100	-	10	Toluene[c]	100	15	< 5	-
6	(BDI)Zn(OAc)	100	-	10	Toluene[b]	50	1	55	29,390 (1.32)

a) Determined by SEC with polystyrene standards and corrected by 0.56[ii], b) [ε-CL]$_0$ = 1 M, c) T = 100 °C and [ε-CL]$_0$ = 8.8 M

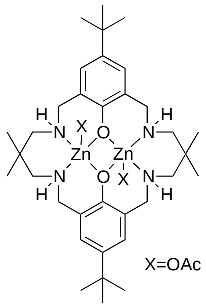

1

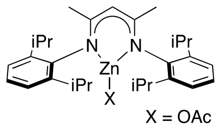

(BDI)Zn(OAc)

Table S2: The formation of polyesters and carbonates: PCHC, PCL and copolymers using catalyst **1**.

Run	CHO	CL	CO_2	t (h)	Polymer	% Conv.[a)] (CL/ CHO)	TON[b)]	TOF[c)] (h^{-1})	M_n[d)] (g/mol)	PDI[d)]
1	-	500	-	16	-	-	-	-	-	-
2[e)]	20	200	-	2	PCL	>99	200	100	11780	1.41
3	900	100	-	2	PCL	>99	200	100	6020	1.24
4	900	100	1	15	PCHC	12	108	7	1040	1.08
5[f)]	900	100	1	21	PCL-PCHC	>99 (CL) 53 (CHO)	577	27	4810	1.28
6[g)]	450	50	1	6.5	PCHC-PCL	6 (CHO); >99 (CL)	77	12	3490	1.48
7	450	50	1	4	PCHC	10 (CHO)	95	13	560	1.29
				2	PCHC-PCL	>99 (CL)			2350	1.49

a) Determined by 1H NMR spectroscopy by integrating the normalised resonances for CL (4.23 ppm) and PCL (4.05 ppm) or CHO (3.11 ppm) and PCHC (4.73 - 4.54 ppm). b) Turn-over-number = # moles monomer(s) consumed/# moles catalyst; for copolymers monomer consumption = CL + CHO. c) Turn-over-frequency = TON/time (h). d) Determined by SEC in THF, with a correction factor of 0.56 applied for PCL samples according to the literature findings, e) Polymerization run in toluene, with [CL]0 = 1M, f) CO_2 added after complete consumption of CL (1 h) as monitored by ATR-IR. g) CO_2 removed after 3.5 h by application of vacuum-nitrogen cycles (6 times).

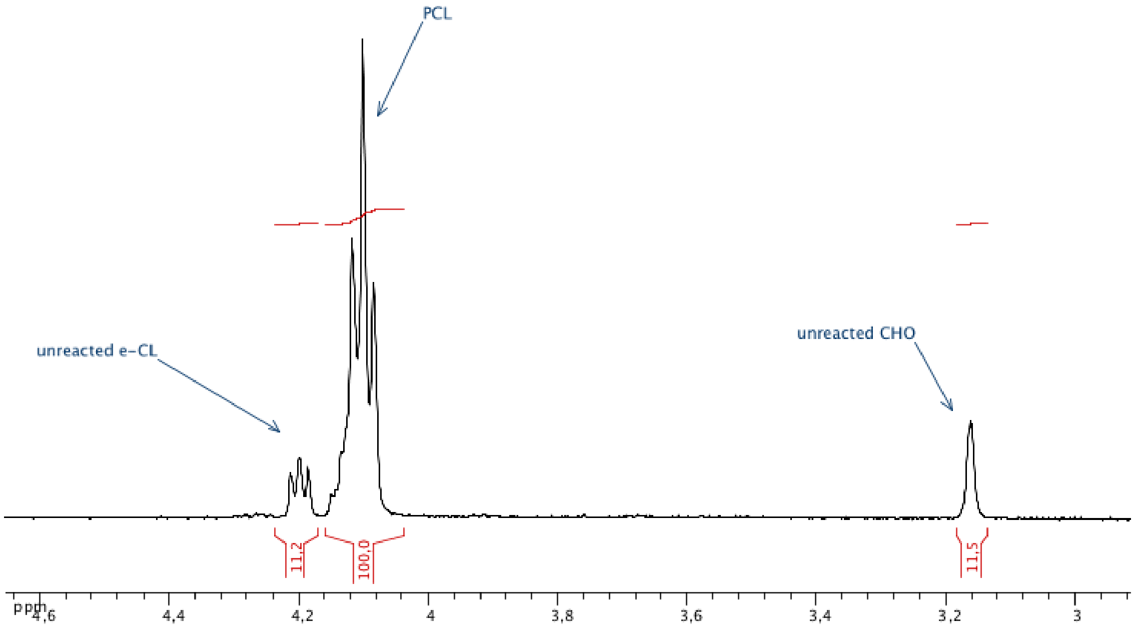

Figure S1: ^{1}H NMR spectrum ($CDCl_3$, 298 K) showing formation of PCL in the presence of CHO, without polymerization of CHO (absence of (poly)ether linkage at 3.45 ppm). Reaction conditions: **1**/CHO/CL = 1/20/200, 2 h, 99 % conv. of CL to PCL (Table S1, Entry 2).

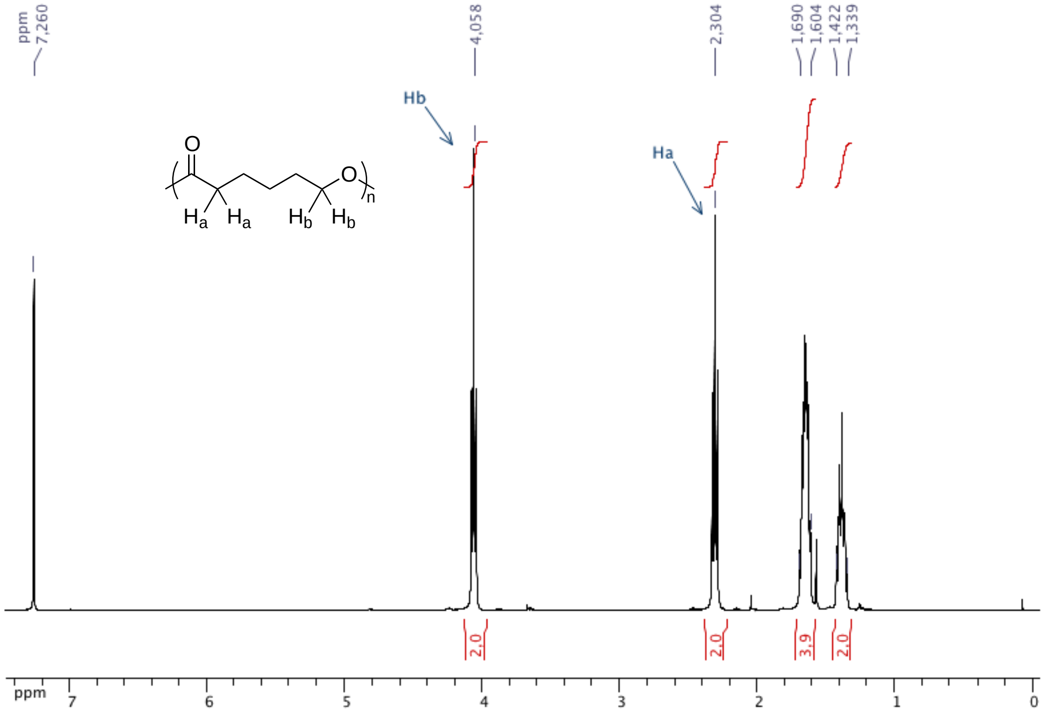

Figure S2: ^{1}H NMR spectrum ($CDCl_3$, 298 K) of an isolated sample of PCL (M_n = 11,780 g.mol^{-1}, PDI = 1.41) from conditions described in Figure S1 (Table S1, Entry 2).

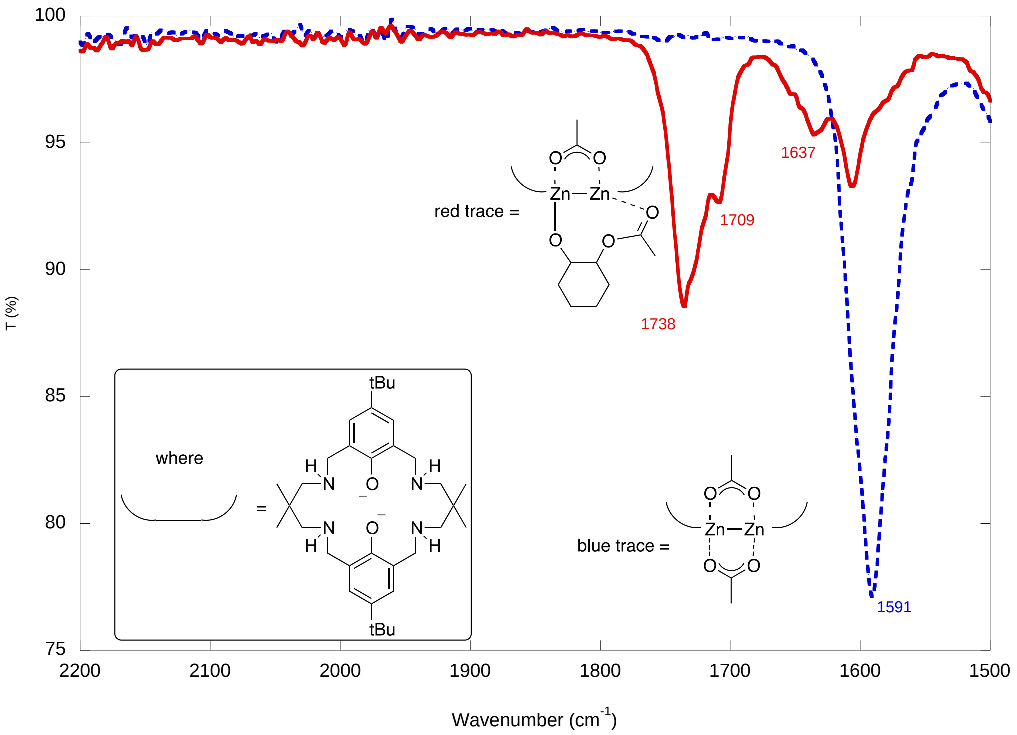

Figure S3: IR spectra of complex **1** (blue line) and **1** after heating at 80 °C for 26 h in the presence of 20 equivalents of CHO, in toluene (red line). The experimental spectra show formation of a new absoption at 1738 cm^{-1}, assigned to both κ^2 and cyclohexene-bound acetate resonance, in line with previous results.[iii]

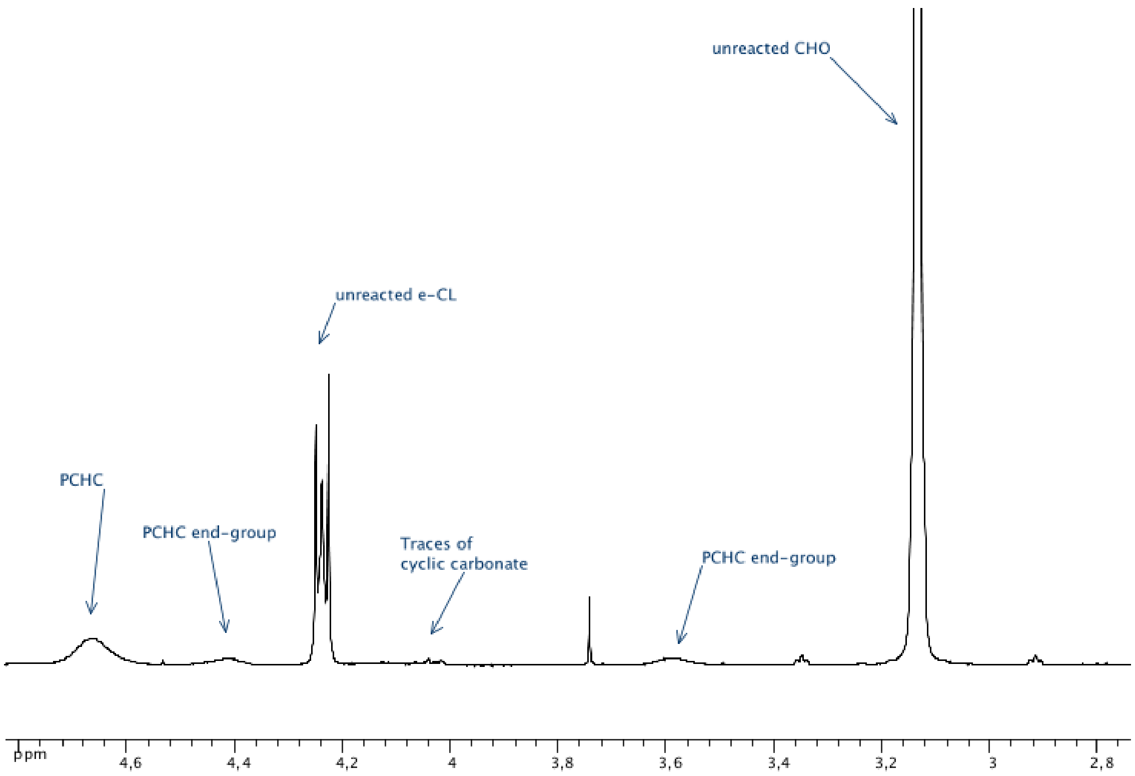

Figure S4: ^{1}H NMR spectrum ($CDCl_3$, 298 K) showing formation of PCHC in the presence of ε-CL, with no formation of PCL (4.00 ppm) or ether linkages (3.45 ppm).

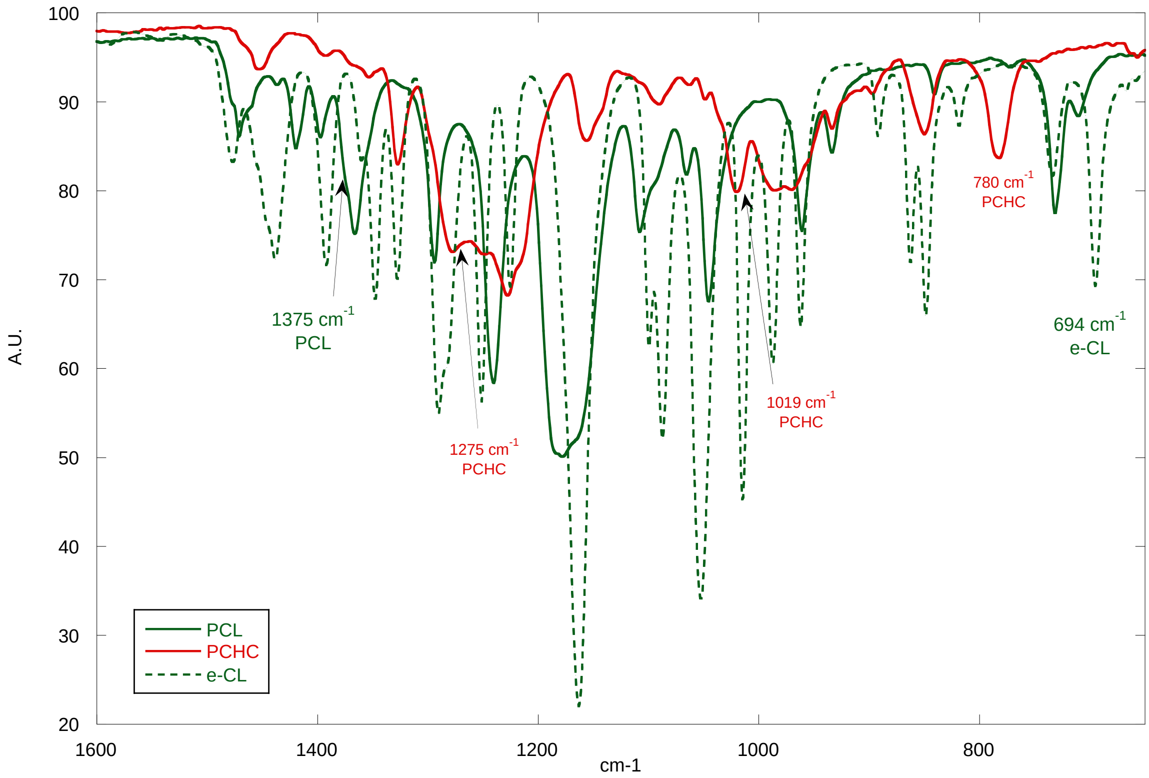

Figure S5: Expanded region of the IR spectra of pure samples of PCHC (red line), PCL (green line) and ε-CL (dashline). The peaks identified represent examples of resonances which can be cleanly assigned to a specific monomer(s) in the mixture.

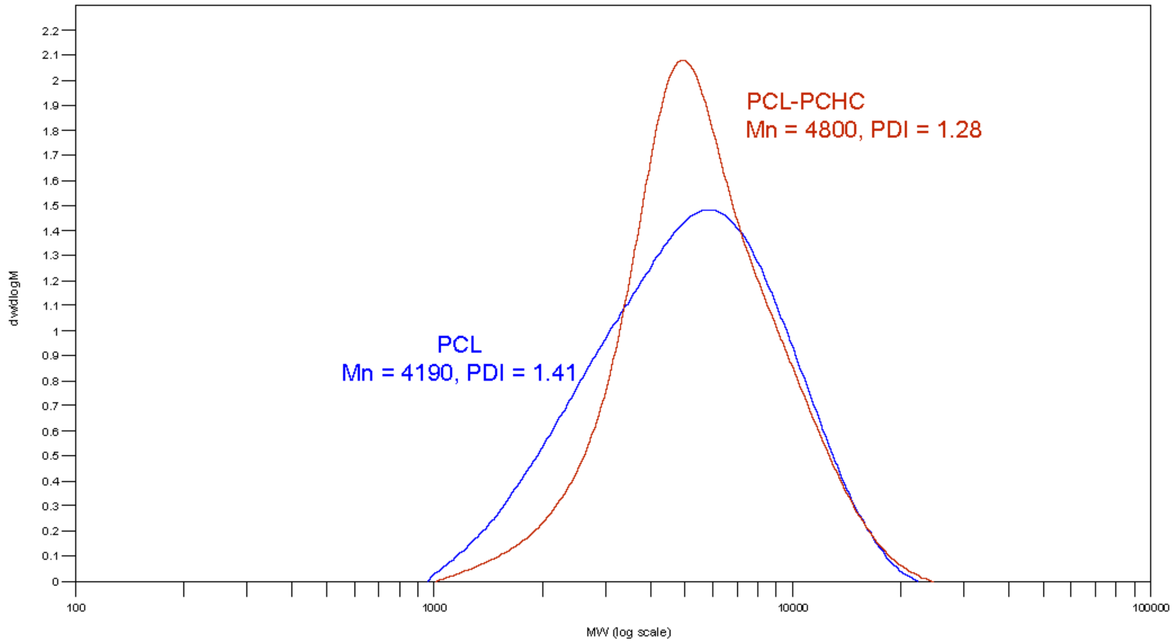

Figure S6: The SEC outputs from the reaction with caprolactone and epoxide, to yield of PCL (blue line), and the product PCL-PCHC, formed after addition of CO_2 (red line) (Table 1, run 5).

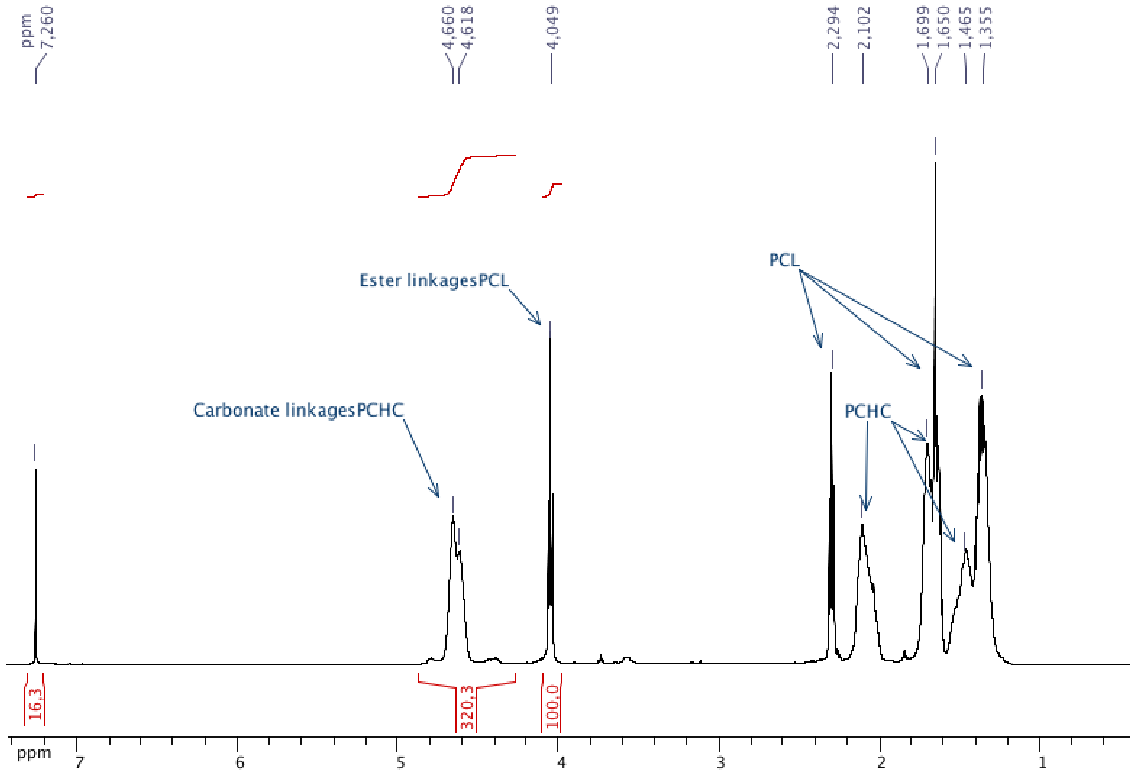

Figure S7: ^{1}H NMR spectrum ($CDCl_3$, 298 K) of the PCL-PCHC copolymer formed under the conditions described in Table 1, run 5. The plot illustrates the formation of both PCHC and PCL blocks.

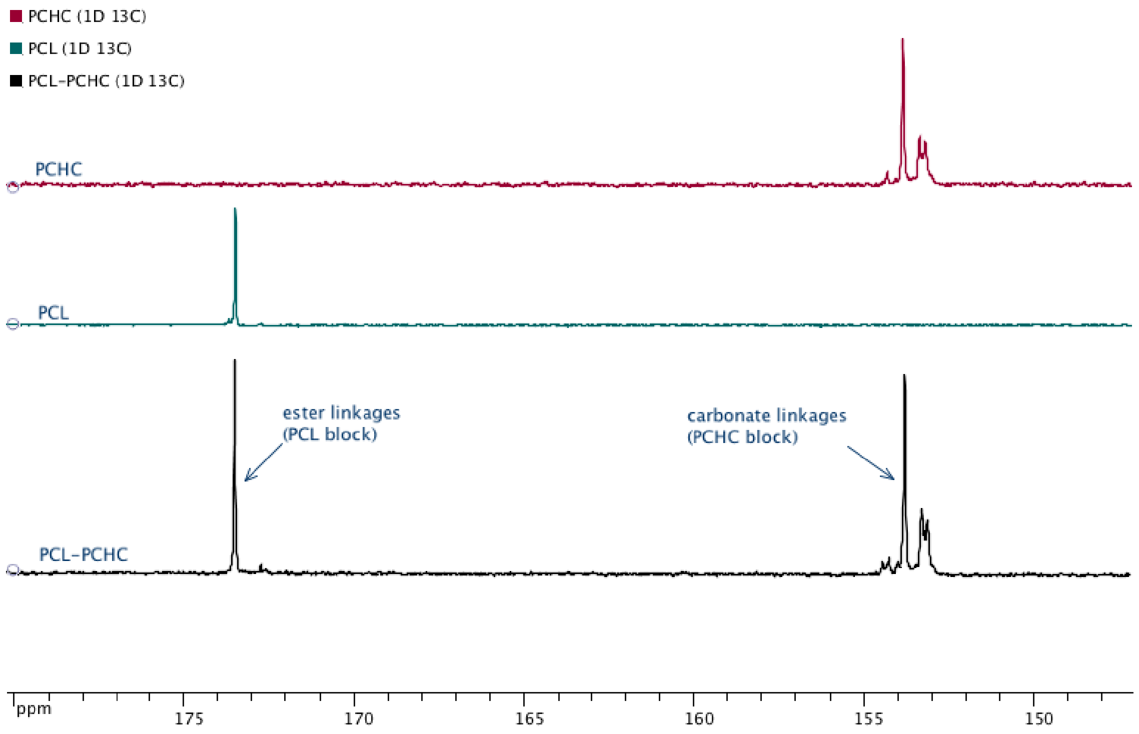

Figure S8: Expanded $^{13}C\{^1H\}$ NMR spectra ($CDCl_3$, 298 K) for the PCL-PCHC copolymer (bottom spectrum, conditions as per Table 1, run 5) compared with pure samples of PCHC (top spectrum) and PCL (middle spectrum), respectively.

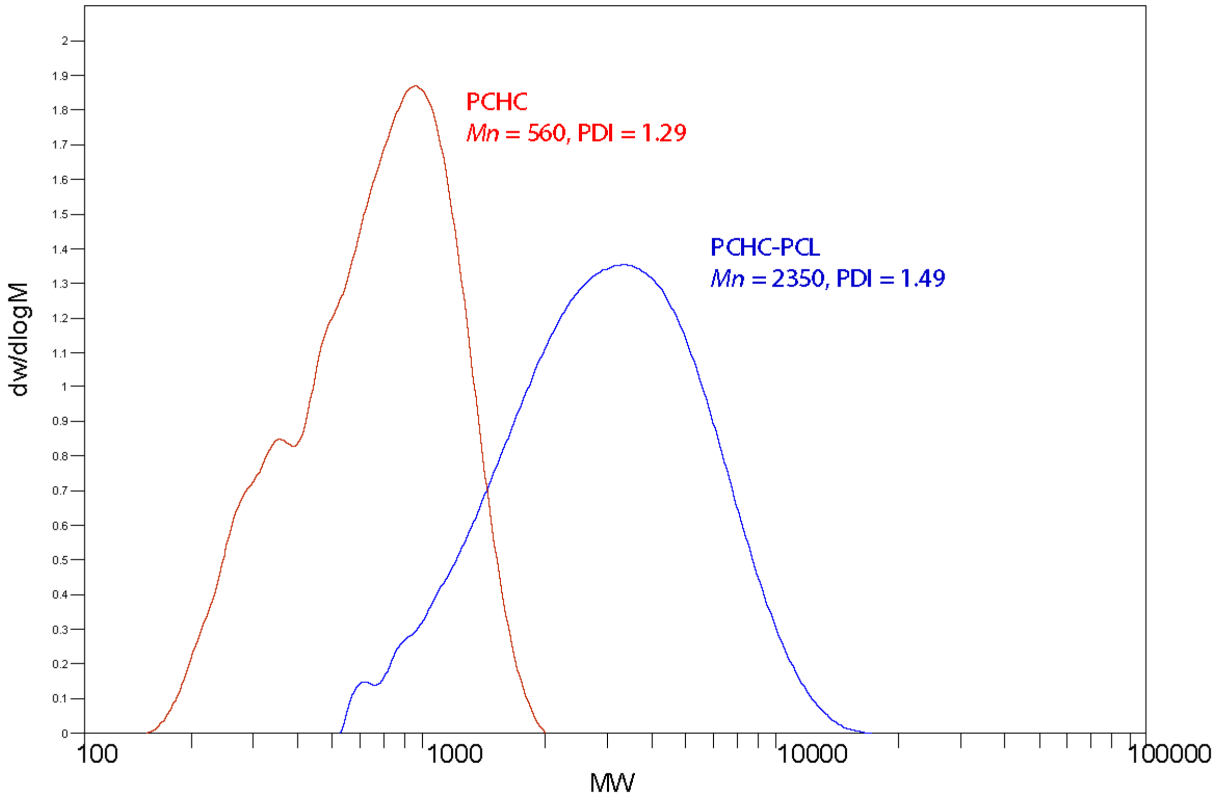

Fig. S9: SEC stack plot showing the analysis of PCHC-PCL formation according to the conditions of Table 1, run 7. The red trace shows the analysis of an aliquot removed after 4 h, which showed 10% CHO conversion and PCHC formation, with M_n 530 g/mol. At this point, the CO_2 was removed leading to CL ROP. After 2 h, a second aliquot was removed was >99% conversion of CL and formation of PCHC-PCL of M_n 2350 g/mol.

---

[i] (a) M. R. Kember, P. D. Knight, P. T. R. Reung, C. K. Williams, *Angew. Chem. Int. Ed.* **2009**, *48*, 931. (b) M. R. Kember, A. J. P. White, C. K. Williams, *Inorg Chem.* **2009**, *48*, 9535 ; (c) M. Cheng, E. B. Lobkovsky, G. W. Coates, *J. Am. Chem. Soc.* **1998**, *120*, 11018-11019.

[ii] M. Save, M. Schappacher, A. Soum, *Macromol. Chem. Phys.* **2002**, *203*, 889-899.

[iii] A. Buchard, F. Jutz, M. R. Kember, A. J. P. White, H. S. Rzepa, C. K. Williams, *Macromolecules* **2012**, *45*, 6781.
